# Huaier aqueous extract protects against dextran sulfate sodium-induced experimental colitis in mice by inhibiting NLRP3 inflammasome activation

**DOI:** 10.18632/oncotarget.16513

**Published:** 2017-03-23

**Authors:** Lijuan Wang, Zhongxia Yu, Chao Wei, Li Zhang, Hui Song, Bing Chen, Qifeng Yang

**Affiliations:** ^1^ Pathology Tissue Bank, Qilu Hospital, Shandong University, Jinan, Shandong 250012, China; ^2^ Department of Immunology and Key Laboratory of Infection and Immunity of Shandong Province, Shandong University School of Medicine, Jinan, Shandong 250012, China; ^3^ Department of Ophthalmology, Second Hospital of Shandong University, Jinan, Shandong 250033, China; ^4^ Department of Breast Surgery, Qilu Hospital, Shandong University, Jinan, Shandong 250012, China

**Keywords:** Huaier, colitis, NLRP3, inflammasome, IL-1β

## Abstract

The use of Trametes robiniophila Murr. (Huaier) as a complementary therapy for cancer has recently become increasingly common in China. However, whether Huaier can regulate host immune responses, especially innate immunity, remains largely unknown. The NLRP3 inflammasome is a multimeric complex consisting of NLRP3, ASC and caspase-1. NLRP3 inflammasomes respond to a variety of endogenous (damage-associated molecular patterns) and exogenous (pathogen-associated molecular patterns) stimuli, and play crucial roles in host defense against pathogens and multiple diseases such as ulcerative colitis (UC). In this study, we investigated the anti-inflammatory effect of Huaier in dextran sulfate sodium (DSS)-induced murine colitis and revealed the underlying mechanisms by targeting NLRP3 inflammasomes. In C57BL/6 mice, oral administration of Huaier attenuated DSS-induced colon shortening and colonic pathological damage. Furthermore, we analyzed the effect of Huaier on NLRP3 inflammasome activation in macrophages. Huaier inhibited NLRP3 inflammasome activation-induced IL-1β secretion and caspase-1 cleavage. Moreover, Huaier decreased NLRP3 protein expression via promoting NLRP3 degradation through the autophagy lysosome pathway. Therefore, our findings demonstrate a novel function for Huaier in the regulation of NLRP3 inflammasome activation and suggest a potential role for Huaier in NLRP3 inflammasome-associated diseases.

## INTRODUCTION

Ulcerative colitis (UC), an idiopathic inflammatory bowel disease characterized by chronic and relapsing inflammation, is a risk factor for colitis-associated colon cancer (CAC) [1]. In the treatment of UC, aminosalicylic acid drugs, hormone drugs, or immunosuppressive agents are often used in western medicine, which can control and relieve symptoms in the short term, but have adverse effects in the long term [2]. Therefore, studies exploring alternative therapies for UC have become a topic of great interest.

In recent years, traditional Chinese medicine (TCM), the most common modality of alternative and complementary treatment, has been established for the treatment of UC [3]. Combination treatments with TCM have shown promising results compared to single conventional treatment for UC [4], indicating that TCM may be a promising alternative treatment for UC in the future.

Trametes robiniophila Murr. (Huaier) is a sandy beige mushroom found on the trunks of trees and has been widely used in TCM for more than 1,600 years [5–7]. Recently, the anticancer effects of Huaier have attracted increasing worldwide interest. Huaier extract has been reported to inhibit the growth of cells from multiple types of cancer, such as hepatocellular carcinoma cells [8, 9], breast cancer cells [5, 10, 11], pulmonary cancer cells [6], ovarian cancer cells [12], etc. The anticancer effect of Huaier is associated with a variety of biological activities, including inhibition of cell proliferation, anti-metastasis, interference with tumor angiogenesis etc. [5–13]. Huaier has also been found to exert its anticancer effect through its tumor-specific immunomodulatory roles. W-NTRP (a neutral water-soluble polysaccharide isolated from Huaier) was reported to prominently stimulate macrophages to produce nitric oxide (NO) through the up-regulation of inducible NO synthase activity in cholangiocarcinoma cells [13]. Li *et al*. also reported that in hepatocellular carcinoma cells, TP-1 (a Huaier polysaccharide) induced an increase in CD4^+^ T cells and decrease in CD8^+^T cells in tumor-bearing mice, and also modulated the release of cytokines, including IFN-γ, IL-2, and IL-10 [8]. Huaier extract was also found to suppress breast cancer via decreasing M2-polarization of macrophages and inhibiting macrophage-induced angiogenesis [11]. Together, these results suggest that Huaier has an immunoregulatory effect. However, whether Huaier can regulate the development of the idiopathic inflammatory bowel disease UC remains largely unknown.

The NLRP3 (NACHT, LRR and PYD domains-containing protein 3) inflammasome is a molecular platform that comprises NLRP3, ASC (apoptosis-associated speck-like protein containing CARD) and caspase-1 (cysteine-requiring aspartate protease-1) [14]. Inflammasome complex assembly can be triggered by specific stimuli from invading pathogens and endogenous “danger signals”, such as nigericin, crystals, extracellular ATP, amyloid-β and alum. Once activated, the cysteine protease caspase-1 can be cleaved to its activated form (p10 and p20) and facilitates the cleavage of pro-IL-1β and pro-IL-18 to mature IL-1β and IL-18 [14]. The NLRP3 inflammasome plays crucial roles in host immune responses against infectious microbes and has been implicated in a host of inflammatory disorders [14–16]. Improper NLRP3 inflammasome activation has been linked to a variety of diseases, such as UC, endotoxin shock, Alzheimer's disease, obesity, atherosclerosis and gout [14–16]. It has been reported that NLRP3 protein expression is a limiting step in inflammasome activation [17]. Thus, regulation of NLRP3 offers an interesting mechanism to alter the inflammatory potential of immune cells and provides a potential target for treatment of NLRP3 inflammasome-associated diseases.

In the present study, we first confirmed that Huaier aqueous extract could regulate host immune responses, especially innate immunity. We then demonstrated that Huaier could inhibit NLRP3 inflammasome activation by promoting NLRP3 protein degradation through the autophagy-lysosome degradation pathway and thereby regulate the development of UC.

## RESULTS

### Huaier treatment ameliorates colitis induced by DSS

**Figure 1:**
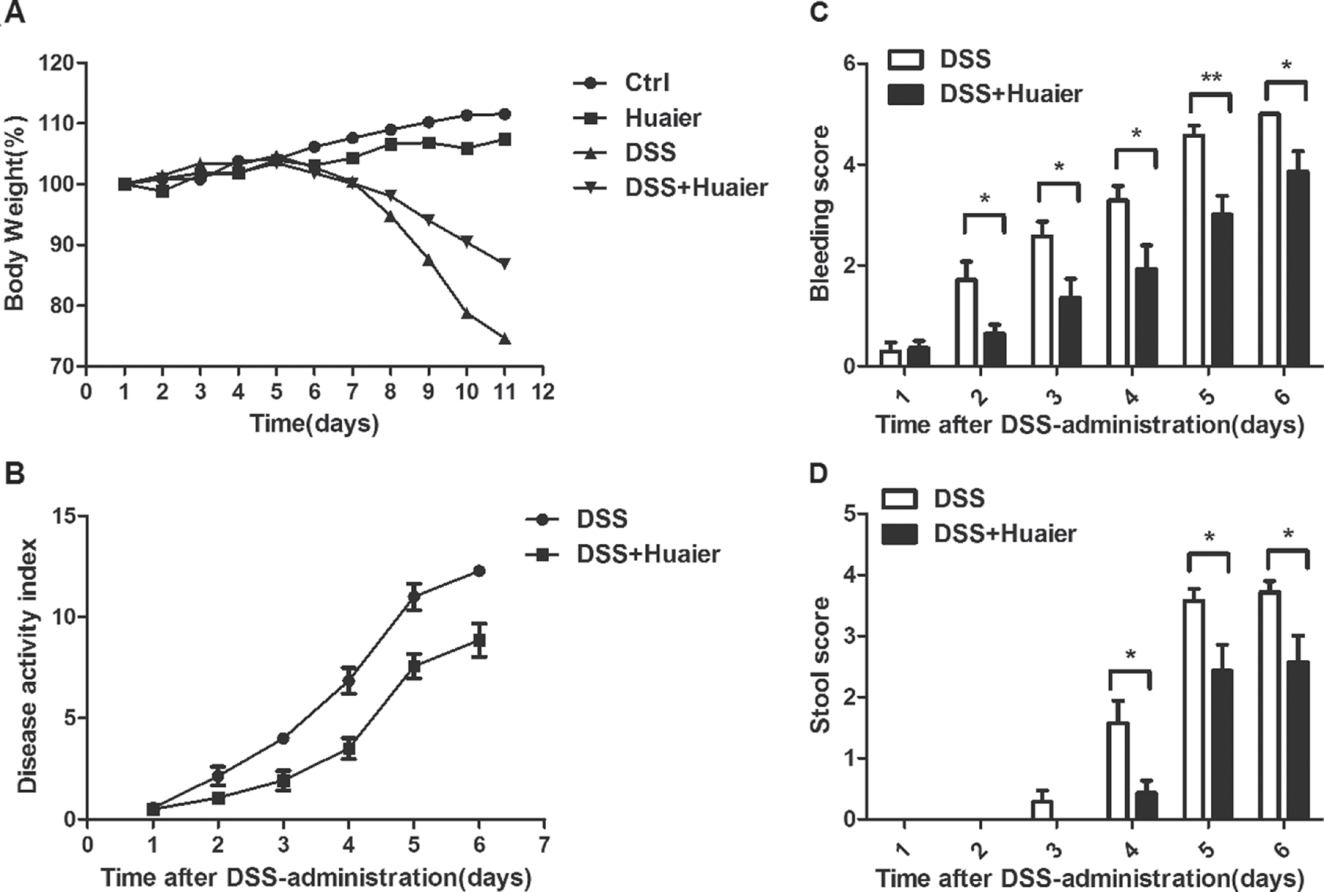
Huaier treatment decreases the susceptibility of mice to DSS-induced experimental colitis Mice were treated with 2.5% DSS in their drinking water for 7 days to induce acute colitis. Every 2 days, each mouse was given 100 μl solution containing 50 mg Huaier extract intragastrically beginning 1 day before DSS administration. (**A**) Body weight and (**B**) Clinical disease activity index were daily observed during the disease process (A: *n* = 8 in control group and Huaier group; *n* = 8 in DSS group and DSS + Huaier group, one-way ANOVA; B: *n* = 8 in DSS group and DSS + Huaier group, Student *t*-test;). (**C**) Bleeding score and (**D**) Stool score of the DSS and DSS+Huaier groups were analyzed during the disease process. Student's *t*-test, *p < 0.05,**p < 0.01. Data are representative of three experiments (mean and s.d. of triplicate samples).

It has been reported that DSS induces severe inflammation in mice with a dramatic loss of body weight [18]. As shown in Figure 1A, DSS-treated mice exhibited profound body weight loss, whereas Huaier could attenuate the loss of body weight significantly. Moreover, the DSS+Huaier-treated group had a much lower disease activity index (DAI) score (Figure 1B), and reduced hemafecia (Figure 1C) and loose stool (Figure 1D) compared with the DSS-treated group after induction of colitis.

### Huaier decreases the DSS-induced histological change in colon

**Figure 2:**
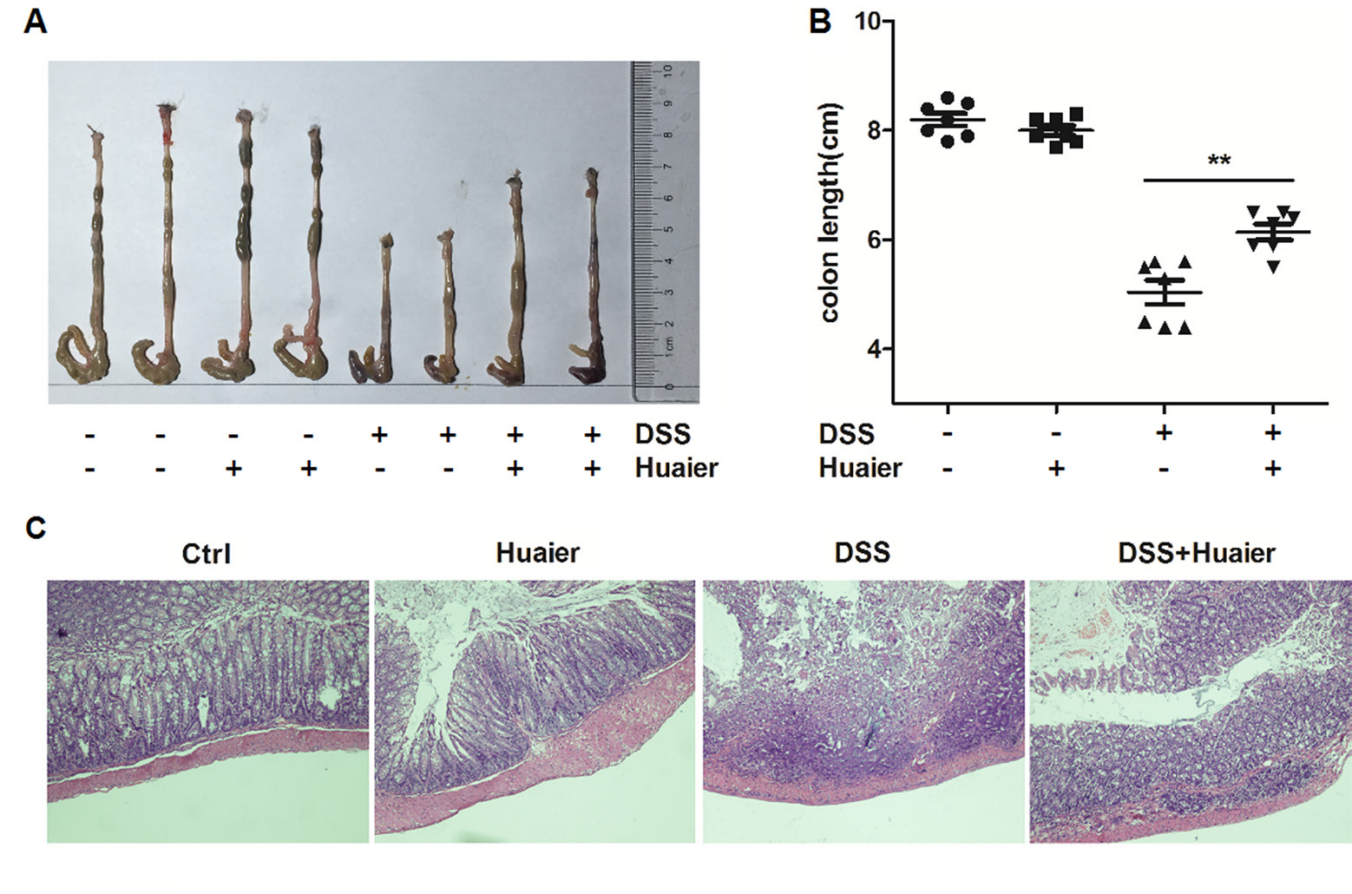
Huaier possesses a protective effect on mice with DSS-induced colitis Mice were sacrificed on day 11 after colitis induction. (**A**) Macroscopic changes and (**B**) colon lengths of the mice were measured. One-way ANOVA, **p < 0.01. Data are representative of three experiments (mean and s.d. of triplicate samples). (**C**) Serial sections of paraffin-embedded colon tissues were stained with H&E. The original amplification was 20×. *n* = 8 in the control and Huaier groups; *n* = 8 in the DSS and DSS + Huaier groups.

To further explore the connection between the clinical signs of colitis and histological parameters, colon tissues were collected from mice. The colon length of DSS-treated mice was obviously shortened compared with the control group. Interestingly, the administration of Huaier significantly attenuated the DSS-induced reduction in colon length (Figure 2A and 2B). Furthermore, the colon tissues were stained with hematoxylin and eosin. Histological results demonstrated severe pathological changes, including loss of goblet cells, distortion of crypts, and infiltration of neutrophils and monocytes, as well as mucosal damage and necrosis in the colon specimens of DSS-treated mice. The administration of Huaier significantly improved these changes (Figure 2C). Collectively, these findings suggest that Huaier treatment ameliorated DSS-induced colitis in mice.

### Huaier aqueous extract inhibits IL-1β secretion

**Figure 3:**
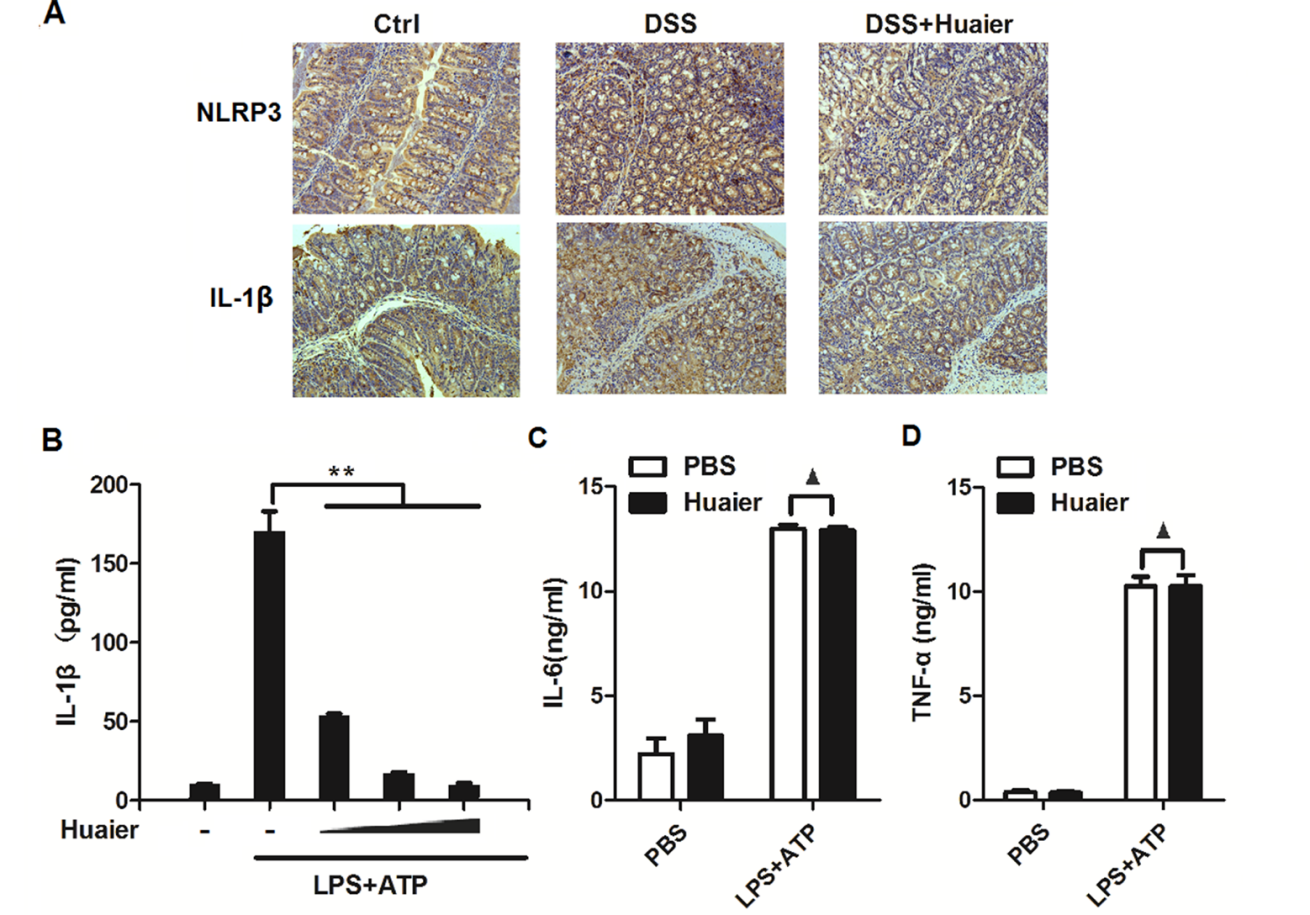
Huaier aqueous extract inhibits IL-1β secretion (**A**) Immunohistochemistry of NLRP3 and IL-1β in colonic tissue section. Positive staining is brown. (**B**) Mouse peritoneal macrophages were pretreated with increasing concentration (0, 4, 8, and 16 mM) of Huaier for 2 h and then primed with LPS (100 ng/ml) for 8 h and stimulated with ATP (2 mM) for 30 min. IL-1β expression in the culture supernatants was determined by ELISA. (**C**) Mouse peritoneal macrophages were pretreated with PBS or Huaier (8 mM) for 2 h and then stimulated as in (B). TNF-α and IL-6 expression in the culture supernatants were determined by ELISA. Student *t*-test, **p < 0.01.▲, no significant differences. Data are representative of three experiments (mean and s.d. of triplicate samples).

It has been documented that the NLRP3 inflammasome plays a critical role in the DSS-induced colitis model [15]. To investigate the mechanism of Huaier-mediated protection from murine colitis, we tested the expression of NLRP3 and IL-1β in colon samples by immunohistochemistry. The results demonstrated that Huaier effectively suppressed NLRP3 and IL-1β expression in the colon specimens of DSS-treated mice (Figure 3A). For further study, IL-1β secretion was examined following Huaier aqueous extract treatment in macrophages. IL-1β secretion was significantly decreased in a dose-dependent manner in Huaier aqueous extract-treated macrophages primed by LPS and then treated by the NLRP3 inflammasome activator, ATP (Figure 3B). However, TNF-α and IL-6 secretion were not influenced by Huaier aqueous extract treatment (Figure 3C and 3D).

### Huaier aqueous extract inhibits NLRP3 inflammasome activation

**Figure 4:**
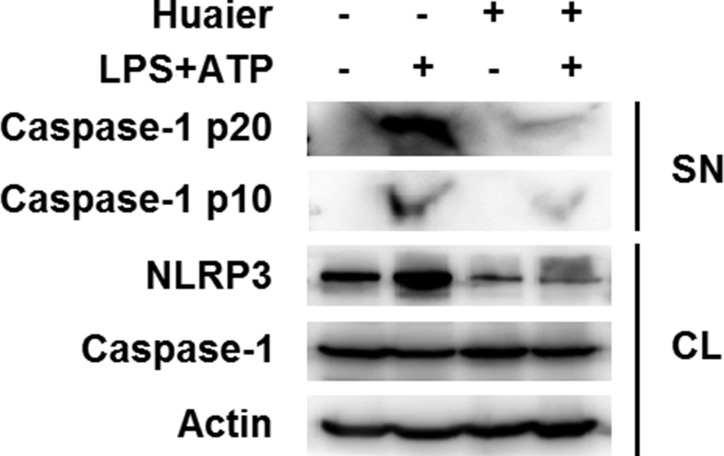
Huaier aqueous extract inhibits caspase-1 cleavage Mouse peritoneal macrophages were pretreated with PBS or Huaier (8 mM) for 2 h and then primed with LPS (100 ng/ml) for 8 h and stimulated with ATP (2 mM) for 30 min. Western blot analysis was of caspase-1 p10 and p20 in the supernatants (SN) and pro-caspase-1 and NLRP3 in cell lysates (CL). Similar results were obtained in three independent experiments.

Caspase-1 cleavage is a critical step for NLRP3 inflammasome activation [14]. We investigated the effects of Huaier aqueous extract on caspase-1 cleavage. Huaier aqueous extract treatment greatly inhibited caspase-1 p10 and p20 expression in the supernatant of LPS-primed mouse peritoneal macrophages treated with ATP (Figure 4). These data indicated that Huaier could inhibit NLRP3 inflammasome activation-induced caspase-1 cleavage and subsequent IL-1β secretion.

### Huaier aqueous extract inhibits NLRP3 protein expression

**Figure 5:**
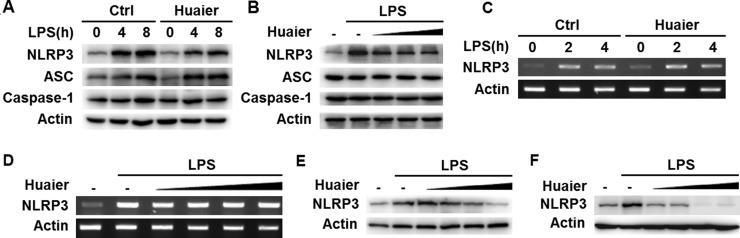
Huaier inhibits NLRP3 protein expression Mouse peritoneal macrophages were pretreated with PBS or Huaier (8 mM) for 2 h and then stimulated with LPS (100 ng/ml) for indicated time periods. NLRP3 expression was detected by Western blot (**A**) or RT-PCR (**C**). Mouse peritoneal macrophages were pretreated with increasing concentration of Huaier (0, 4, 8, 16 mM for B, and 0, 2, 4, 8, 16 mM for D) for 2 h and then stimulated with LPS (100 ng/ml) for 4 h. NLRP3 expression was detected by Western blot (**B**) or RT-PCR (**D**). RAW264.7 (**E**) and THP-1 cells (**F**) were pretreated with PBS or Huaier (0, 2, 4, 8, 16 mM for E, and 0, 4, 8, 16 mM for F) for 2 h and then stimulated with LPS for 4 h. NLRP3 expression was detected by Western blot. Similar results were obtained in three independent experiments.

It has been reported that Huaier possesses anticancer activity by inhibiting the expression of multiple molecules, such as MMP2, MMP9 and VEGF [11]. We then investigated whether Huaier could regulate expression of key molecules in the NLRP3 inflammasome complex. Huaier aqueous extract treatment greatly inhibited NLRP3 expression at the protein level in mouse peritoneal macrophages, with no influence on ASC and caspase-1 expression (Figure 4 and Figure 5A). Furthermore, Huaier aqueous extract could inhibit NLRP3 protein expression in a dose-dependent manner (Figure 5B). Interestingly, Huaier aqueous extract treatment had no effect on NLRP3 mRNA expression (Figure 5C and 5D). Consistent with previous experiments, Huaier aqueous extract greatly inhibited NLRP3 protein expression in a dose-dependent manner in mouse RAW264.7 cells and human THP-1 cells (Figure 5E and 5F). Taken together, these data indicated that Huaier could inhibit NLRP3 expression at the protein level.

### Huaier aqueous extract inhibits NLRP3 expression by promoting its lysosome-dependent degradation

**Figure 6:**
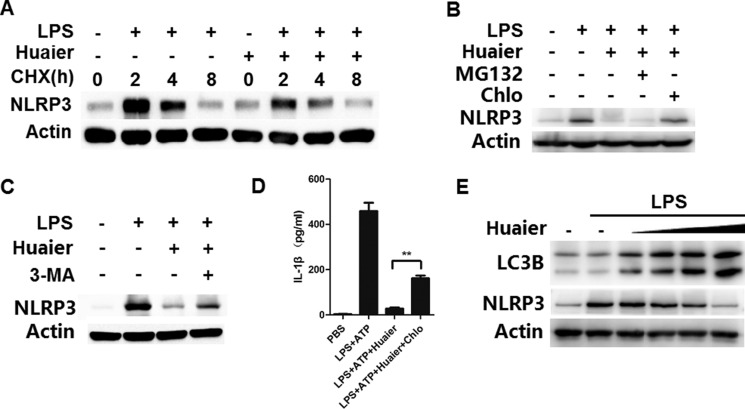
Huaier promotes NLRP3 degradation via promoting autophagy (**A**) Mouse peritoneal macrophages were pretreated with Huaier (8 mM) for 2 h, then stimulated with LPS for 4 h, and subsequently treated for various times with cycloheximide (CHX) (10 μM). NLRP3 expression levels were detected by Western blot. (**B**, **C**) Mouse peritoneal macrophages were pretreated with Huaier (8 mM) for 2 h and then stimulated with LPS (100 ng/ml) for 8 h. The cells were treated with MG132 (10μM), chloroquine (100 μM) (B) and 3-MA (10 mM)(C) for the last 4 h. NLRP3 expression was detected by Western blot. (**D**) Mouse peritoneal macrophages were pretreated with Huaier (8 mM) for 2 h, primed with LPS (100 ng/ml) for 4 h, treated with chloroquine (100 μM) and finally stimulated with ATP (2 mM) for 30 min. IL-1β expression in the culture supernatants was determined by ELISA. (**E**) Mouse peritoneal macrophages were pretreated with increasing concentration of Huaier (0, 2, 4, 8, and 16 mM) for 2 h and then stimulated with LPS (100 ng/ml) for 8 h. LC3B and NLRP3 were detected by Western blot. Similar results were obtained in three independent experiments.

We next explored the mechanisms by which Huaier inhibited NLRP3 protein expression. We used cycloheximide to inhibit protein translation of NLRP3, but Huaier could still inhibit NLRP3 expression at the protein level (Figure 6A). We inferred that Huaier might inhibit NLRP3 protein expression via promoting its degradation. Two major protein degradation pathways, the ubiquitin-proteasome pathway and the autophagy-lysosome pathway, are critical for maintaining protein expression levels. We then investigated whether Huaier could promote NLRP3 degradation through the ubiquitin-proteasome or autophagy-lysosome pathways. As shown in Figure 6B and 6C, Huaier aqueous extract-induced NLRP3 degradation could be reversed by the autophagy inhibitors chloroquine and 3-MA, but not by the proteasome inhibitor MG132, indicating that Huaier promoted NLRP3 degradation in lysosomes. When autophage-lysosome degradation of NLRP3 was inhibited by chloroquine, the suppression of IL-1β expression by Huaier was reversed (Figure 6D). Furthermore, significant accumulation of LC3B-II was observed in Huaier aqueous extract-treated macrophages (Figure 6E), indicating that Huaier could promote autophagy. Accordingly, NLRP3 protein expression was greatly decreased following Huaier aqueous extract treatment (Figure 6E). Collectively, these data indicate that Huaier decreases NLRP3 protein expression by promoting its degradation via the autophagy-lysosome pathway.

## DISCUSSION

Inflammation has long been proposed to increase the risk of developing different types of cancer including bladder, cervical, gastric, intestinal, esophageal, ovarian, prostate and thyroid tumors. Inflammatory cells, which are involved in the tumor microenvironment, play an indispensable role in the occurrence, development and promotion of cancer. For example, UC contributes to the high risk of colon cancer [19]. Recently, it was reported that there are various evolving therapeutic options for UC. Immunosuppressive drugs such as TNF-α antibodies [20], AZA (azathioprine) and MTX (methotrexate) [21] have been adopted to control symptoms. However, these immunosuppressants have limitations in efficacy and safety [22].

TCM is the traditional Chinese clinical practice of using plants or/and plant extracts for medical treatment. Currently, increasing numbers of patients are using TCM for disease conditions such as headache and infections. Approximately 9.6% to 12.1% of US adults use one or more forms of TCM to alleviate disease symptoms, including approximately 10% who use TCM for digestive symptoms [23]. Recently, TCM has been included in clinical trials for UC treatment in many countries including China and India [19]. However, the clinical trials were conducted in a small number of UC patients, and the detailed mechanisms of TCM are still poorly understood.

Huaier, one of the most popular medical fungi in China, belongs to the Polyporaceae family and has been used as a TCM for almost 1,600 years [24]. Previous publications have reported Huaier's multiple anticancer effects in hepatocellular carcinoma [8, 9], breast cancer [5, 10, 11], pulmonary cancer [6], ovarian cancer [12], etc. but not in inflammation. Generally, DSS-induced colitis is established to evaluate animals’ susceptibility to intestinal inflammation [25]. Therefore, we evaluated the efficacy of Huaier on DSS-induced colitis. In this study, Huaier ameliorated signs and symptoms of colonic inflammation induced by DSS. We found that Huaier prevented DSS-induced colonic pathological damage as well. Hence, Huaier might be a promising candidate for the treatment of colitis.

Increased pro-inflammatory cytokine production is a hallmark of DSS-induced colitis [26] and among these cytokines, IL-1β plays an important role in intestinal inflammation [27]. In our study, Huaier aqueous extract successfully decreased the high-production of IL-1β induced by LPS and ATP in a dose-dependent manner in macrophages. Meanwhile, TNF-α and IL-6 secretion were not influenced. Generation of IL-1β requires the activation of caspase-1 which converts pro-IL-1β into its mature active form depending on activated inflammasomes. Therefore, inflammasomes are believed to mediate host defenses against microbial pathogens and maintain intestinal homeostasis and thereby contribute to inflammatory diseases and colon cancer [28]. Although there are many types of inflammasomes, the NLRP3 inflammasome is the most extensively studied and the most complicated caspase-1 inductor in intestinal inflammation and colonic neoplasia [29].

NLRP3 is the key component of the NLRP3 inflammasome, and its expression is the rate-limiting step of NLRP3 inflammasome activation [14, 17]. Thus, its expression must be tightly regulated. Previously, it has been reported that the aryl hydrocarbon receptor (AhR) can bind to the NLRP3 promoter and inhibit its expression at the transcriptional level [30] and that the autophagy-lysosome protein degradation pathway is involved in the regulation of NLRP3 protein expression [31, 32]. Plasminogen activator inhibitor type 2 (PAI-2) enhances NLRP3 degradation in an autophagy-dependent manner [31]. Dopamine D1 receptor (DRD1) signaling promotes NLRP3 ubiquitination via the E3 ubiquitin ligase MARCH7, leading to autophagy-mediated degradation of NLRP3 [32]. In this study, we showed that Huaier could inhibit NLRP3 expression at the protein level by promoting its degradation through the autophagy-lysosome pathway.

In conclusion, our study has demonstrated a novel function for Huaier in the regulation of NLRP3 inflammasome activation by promoting NLRP3 protein degradation and thereby regulating the development of UC. Given the pathological role of NLRP3 inflammasomes in UC, Huaier may have therapeutic potential to treat UC characterized by improper NLRP3 inflammasome activation.

## MATERIALS AND METHODS

### Mice and cells

C57BL/6 mice were obtained from Vital River Laboratory Animal Technology Company (Beijing, China). All animal experiments were undertaken in accordance with the National Institute of Health Guide for the Care and Use of Laboratory Animals, with the approval of the Scientific Investigation Board of Shandong University School of Medicine, Jinan, Shandong Province, China. Mouse primary peritoneal macrophages were prepared as described [30, 33]. Mouse macrophage cell line RAW264.7 and human THP-1 cells were obtained from American Type Culture Collection (Manassas, VA). The cells were cultured in endotoxin-free DMEM with 10% (vol/vol) fetal bovine serum (Invitrogen-Gibco). Phorbol myristate acetate (PMA)-activated THP-1 cells were used as human macrophages.

### Reagents

Huaier aqueous extract was obtained and prepared as described [24]. DSS (5000 kDA) was from Wako Pure Chemical Industries(Osaka, Japan); ATP, chloroquine, PMA and LPS (Escherichia coli, 055:B5) were from Sigma-Aldrich(St. Louis, MO, USA); MG132 was from Merck-Millipore(Temecula, CA, USA); 3-MA was from Selleck Chemicals(Houston, TX, USA) ; cycloheximide(CHX) was from ApexBio(Houston, TX, USA); anti-NLRP3 Ab and anti-ASC Ab, anti-caspase-1 p10 Ab and p20 Ab were from Adipogen(San Diego, CA, USA); anti-LC3B Ab was from Cell Signaling Technology(Trask Lane Danvers, MA, USA); anti-IL-1β was from Novus Biologicals(Littleton, CO, USA).

### DSS-induced colitis and design of drug treatment

Six week old female C57/BL6 mice, weighing 19–22 g, were supplied by Vital River Laboratory Animal Technology Company (Beijing, China). Experimental protocols were in accordance with National Institutes of Health regulations and approved by the Institutional Animal Care and Use Committee. Throughout the acclimatization and study periods, all animals had access to food and water ad libitum and were maintained on a 12h light:12h dark cycle (21 ± 2°C with a relative humidity of 45 ± 10°C). Acute colitis was induced by administration of DSS in drinking water. The mice were randomly assigned to control, DSS-treated, Huaier-treated and DSS+Huaier-treated groups. The mice received either normal drinking water (control) or 2.5% (w/v) DSS drinking water (model) for 7 days (day 2 to day 8) and normal water for the following 3 days (day 9 to day 11) [34]. Each mouse was given 100 μl of solution containing 50 mg Huaier extract intragastrically every 2 days (day 1 to day 11) [24].

### Clinical scoring and histological analysis

Mice were monitored for body weight, stool consistency and the presence of gross blood in feces and at the anus every day. The Assessment of Disease Activity Index (DAI) was the sum of the scores given for “weight loss,” “stool consistency,” and “occult/gross bleeding.” The definitions and scores for these parameters were (0) no weight loss with normal stool consistency, no occult/gross bleeding was observed; (1) 1 to 5% weight loss with loose stools and positive fecal occult test; (2) 5 to 10% weight loss with loose stools and positive fecal occult test; (3) 10 to 20% weight loss with diarrhea and gross bleeding; and (4) more than 20% weight loss with diarrhea and gross bleeding [35]. On day 11 following induction of colitis, all mice were euthanized. Colons were quickly and safely removed and washed with PBS, and the lengths of colons were measured. For histological analysis, formalin-fixed and paraffin-embedded segments of colon tissue were sectioned at 4 μm in thickness and stained with hematoxylin and eosin (H&E) [36].

### Immunohistochemistry

Immunohistochemistry assay was performed as described in [34]. Briefly, paraffin-embedded slides were deparaffinized, rehydrated and washed in 1% PBS-Tween. They were then treated with 3% hydrogen peroxide and blocked with 10% goat serum for 1h at 37°C. Slides were incubated with primary antibodies in PBS containing 1% BSA (1:200) for 1h at 37°C. Biotinylated secondary anti-rabbit (or mouse) antibodies were added and incubated at room temperature for 1 h. Streptavidin-HRP was added, and after 40 min, the sections were stained with DAB substrate and counterstained with hematoxylin.

### ELISA

The concentrations of IL-1β, TNF-α and IL-6 were measured using an ELISA kit (Dakewe Biotech Company Ltd., Shenzhen, China) according to the manufacturer's instructions [30].

### RT-PCR

Total RNA was extracted with RNAiso Plus reagent according to the manufacturer's instructions (TaKaRa). The sequences of primers used for RT-PCR were 5′-TGGATGGGTTTGCTGGGAT-3′ and 5′-CTGCGTGTAGCGACTGTTGAG-3′ for NLRP3; 5′-TGTTACCAACTGGGACGACA-3′ and 5′-CTGGGT CATCTTTTCACGGT-3′ for β-actin.

### Western blot

Cells were lysed with M-PER Protein Extraction Reagent (Pierce, Rockford, IL) supplemented with a protease inhibitor “cocktail,” then protein concentrations in the extracts were measured with a bicinchoninic acid assay (Pierce, Rockford, IL). Supernatants from cell culture were collected and concentrated for Western blot analysis with Amicon Ultra 10K from Millipore [30]. Equal amounts of extracts were separated by SDS-PAGE and then transferred onto nitrocellulose membranes for immunoblot analysis [30, 33].

### Statistical analysis

All experiments were independently performed three times. Data are presented as the means ± S.D. of three or four experiments. Analysis was performed using one-way ANOVA and Student's *t*-test. Values of *p* < 0.05 were considered to be statistically significant.

## Abbreviations

UC: ulcerative colitis
CAC: colitis-associated colon cancer
DSS: dextran sulfate sodium
TCM: traditional Chinese medicine
NLRP3, NACHT: LRR and PYD domains-containing protein 3
ASC: apoptosis-associated speck-like protein containing CARD
ATP: adenosine triphosphate
